# Risk of Incident Epilepsy After a Middle Cerebral Artery Territory Infarction

**DOI:** 10.3389/fneur.2022.765969

**Published:** 2022-03-03

**Authors:** Cheng-Yang Hsieh, Chien-Chou Su, Edward Chia-Cheng Lai, Yu-Shiue Chen, Tzu-Hsin Huang, Yea-Huei Kao Yang, Chih-Hung Chen, Sheng-Feng Sung, Chin-Wei Huang

**Affiliations:** ^1^Department of Neurology, Tainan Sin Lau Hospital, Tainan, Taiwan; ^2^School of Pharmacy, Institute of Clinical Pharmacy and Pharmaceutical Sciences, College of Medicine, National Cheng Kung University, Tainan, Taiwan; ^3^Department of Neurology, National Cheng Kung University Hospital, College of Medicine, National Cheng Kung University, Tainan, Taiwan; ^4^Division of Neurology, Department of Internal Medicine, Ditmanson Medical Foundation Chia-Yi Christian Hospital, Chiayi City, Taiwan

**Keywords:** middle cerebral artery, seizure, epilepsy, stroke, infarct

## Abstract

**Background:**

Among poststroke morbidities, poststroke epilepsy (PSE) has been identified as a significant clinical issue. Although middle cerebral artery (MCA) infarct is the most common type of stroke among all vascular territories, very few studies specifically focused on the risk factors leading to PSE in patients with MCA infarct.

**Methods:**

A population study in Taiwan has been conducted, linking the National Health Insurance Research Database and Hospital Stroke Registry, from 2001 to 2015 and 2006 to 2010, respectively. Patients were divided into MCA and non-MCA groups, and the diagnosis of incident epilepsy between the groups has been compared. The multivariable Cox proportional hazard model was used to identify the risk factors for developing PSE. The distribution of time to PSE was estimated using the Kaplan–Meier method.

**Results:**

In total, 1,838 patients were recruited, with 774 and 1,064 in the MCA and non-MCA groups, respectively. PSE incidence in the MCA group was 15.5% vs. 6.2% in the non-MCA group, with a hazard ratio of (95% CI) 2.06 (1.33–3.19). Factors significantly associated with PSE included atrial fibrillation, depression, National Institutes of Health Stroke Scale (NIHSS) scores of ≥ 16, and alert on arrival. For patients with MCA infarct, higher NIHSS and Glasgow coma scale scores, the presence of visual field defects and weakness, urination control impairment, and complications during hospitalization were associated with a higher risk for PSE development.

**Conclusions:**

This study established the conditions leading to a higher risk of PSE and identified the important clinical risk factors in patients experiencing MCA infarct. Efforts to manage these risk factors may be important in preventing PSE in patients with MCA infarct.

## Introduction

Advances in stroke treatment resulted in a dramatic reduction in stroke mortalities; however, the number of stroke survivors living with morbidities increased significantly. Among these morbidities, seizures and epilepsy are not uncommon, and PSE has been identified as a significant clinical issue among stroke survivors. As we know, stroke is the most common cause of epilepsy in older adults and for patients aged more than 65 years, where PSE accounts for 30–50% of new-onset seizures (1). The incidence of early seizures (occurring within the first 1–2 weeks after a stroke) is between 2.4 and 5.4%, and the risk of poststroke late seizures (seizures occurring later than 14 days after a stroke) is around 7–18% (1, 2). Our previous study documented that seizures at stroke presentation and during hospitalization worsen overall morbidity and mortality, (3) suggesting the importance of awareness of seizure care in the treatment of ischemic stroke.

The stroke severity, location, and type of pathological changes, genetic factors, and preinjury and postinjury exposure to nongenetic factors including exposome can be used and divide patients experiencing an ischemic stroke into different levels of susceptibility (4–6), where the standardized rate of developing epilepsy is highest during the first year (7). Studies showed that a higher National Institutes of Health Stroke Scale (NIHSS) score, cortical involvement at a younger age, and central nervous system morbidities are associated with a higher risk of PSE (1). Moreover, ethnicity may play an important role in the development of PSE. For example, the prevalence of poststroke seizures in patients without atrial fibrillation is higher in Australia than it is in China (8).

The middle cerebral artery (MCA) infarct is the most common form of stroke among all vascular territories (9). The cerebral cortex and regions in the MCA territory, i.e., the temporal and frontal lobes, may be more susceptible to epileptogenesis after a stroke than other brain areas (10). Surprisingly, the risk factors of epilepsy after an MCA infarct are not clearly known and have not been specifically addressed. Although one study reported a higher incidence of seizures in an MCA group than in an internal carotid artery group (11) and one study reported MCA infarct as one of the main variables associated with PSE risk, (12) no study was found specifically focusing on post-MCA infarct epilepsy and also comparing this group of patients with patients experiencing non-MCA strokes. Furthermore, the interaction between an MCA infarct and other risk factors has not been thoroughly explored.

This study conducted a large-scale epidemiological study, linking a large dataset stroke registry and an administrative claims database to investigate the incidence and risk factors related to post-MCA infarct epilepsy in Taiwan to unravel the potential clinical risk factors and make a specific comparison with post-non-MCA infarct epilepsy.

## Methods

### Data Source and Linkage

Two databases have been linked for this study: the National Health Insurance Research Database (NHIRD) from 2001 to 2015 and the Hospital Stroke Registry from 2006 to 2010. The NHIRD is derived from the National Health Insurance program, which covers nearly all of the Taiwanese population. It includes baseline demographics for beneficiaries, outpatient care claims, inpatient claims, and claims for medication dispensed at pharmacies. Each claim contains the International Classification of Diseases, Ninth Edition, Clinical Modification (ICD-9-CM) diagnosis and procedure codes, expenditures per visit or admission, and details of drug prescriptions. The NHIRD used in this work was provided by the Ministry of Health and Welfare and is maintained by the Health and Welfare Data Science Center (HWDC) in Taipei City, Taiwan [https://nhird.nhri.org.tw/en/]. For further details of Taiwan's NHIRD, readers can refer to Hsieh et al. (13).

The Hospital Stroke Registry database in this study was derived from two Taiwan Stroke Registry (TSR) participating hospitals, i.e., the National Cheng Kung University Hospital and Ditmanson Medical Foundation Chiayi Christian Hospital. The TSR, launched in August 2006, is a nationwide stroke registry used to collect data on the quality of stroke care and to inform healthcare policy making (14, 15). The ongoing TSR enrolls patients who are hospitalized for acute stroke or transient ischemic attacks within 10 days of symptom onset. By 2015, >100,000 stroke events have been registered in the TSR (16).

The two databases were linked by the national identification number in the HWDC to ensure the security of personal information, so the researchers could only access the linked databases in the HWDC (Supplementary Figure 1). Because only aggregate data without any individual-level information can be released from the HWDC, informed consent from individual patients were not required. The research protocol was approved by the Institutional Review Board of National Cheng Kung University Hospital (IRB No. A-ER-108-563).

### Study Population

**Figure 1:**
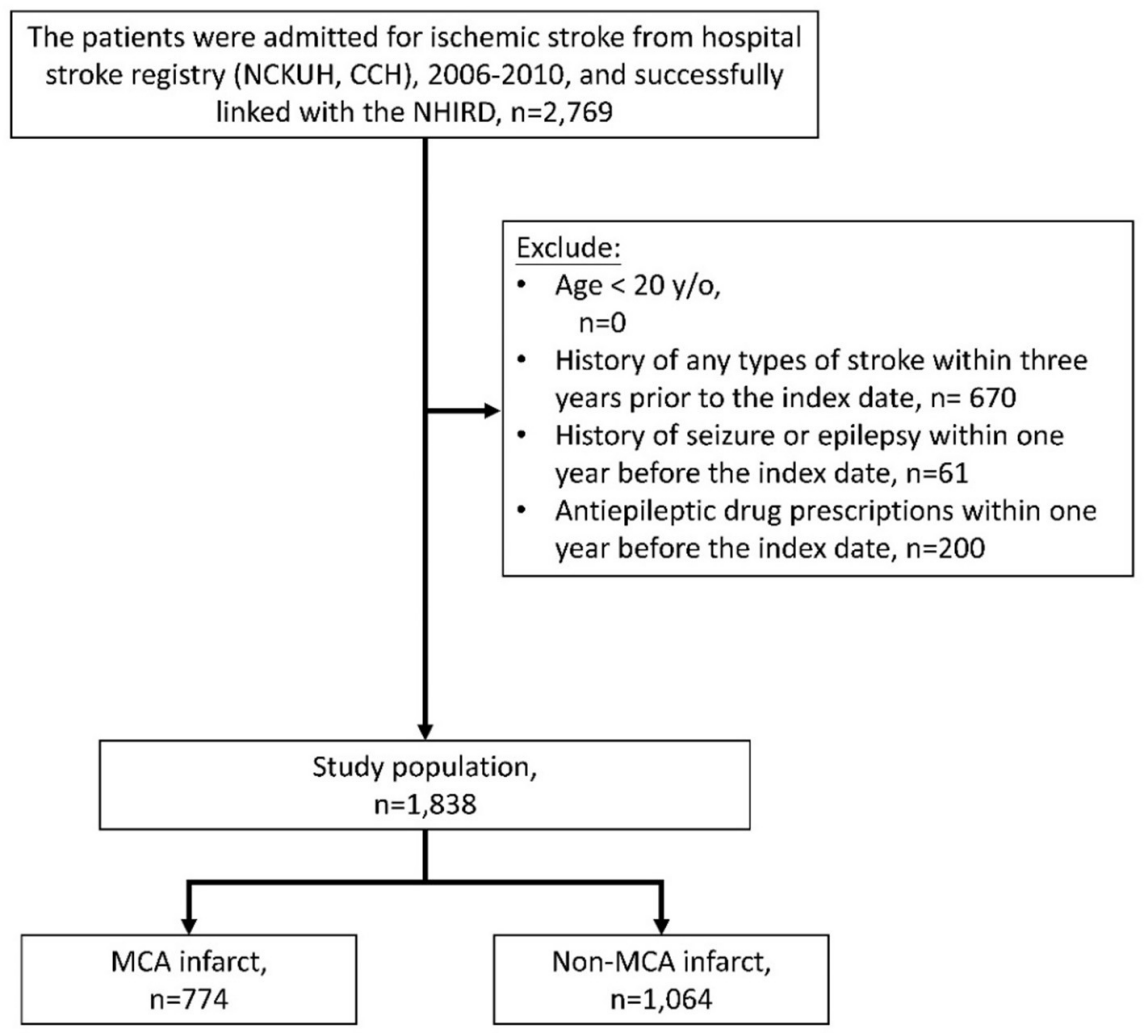
Collection of the study population. NCKUH, National Cheng Kung University Hospital; CCH, Chia-Yi Christian Hospital.

Patients hospitalized for acute ischemic stroke (ICD-9-CM codes 433 or 434) who survived and were discharged between 2006 and 2010 were identified from the Hospital Stroke Registry (Figure 1). After data linkage with the NHIRD, claims records of successfully linked patients were retrieved. For each patient, the successfully linked stroke hospitalization episode was defined as the index stroke hospitalization, and the discharge date of the index stroke hospitalization was defined as the index date. Patients who were younger than 20 years old were excluded. To ensure that it was a first-ever stroke, patients who demonstrated claims of any type of stroke (ICD-9-CM codes 430–438) within 3 years before the index date were excluded. Patients who demonstrated claims for seizure or epilepsy (ICD-9-CM codes 7803 or 345) or who received antiepileptic drugs within 1 year before the index date were also excluded.

### Main Independent Variable and Covariates

The main independent variable of interest was the presence of an MCA territory infarction. According to the infarct location recorded in the stroke registry, patients were divided into MCA and non-MCA cohorts, respectively. From the dataset linking the claims data and stroke registry, baseline demographics, stroke severity on admission, functional status at discharge, laboratory data, comorbidities, medication uses, presence of complications, and neurosurgery records for the index stroke hospitalization have been retrieved. Stroke severity was represented by the NIHSS score on initial presentation, while functional status was represented with the modified Rankin Scale (mRS) and Barthel Index scores at discharge, as recorded in the Hospital Stroke Registry.

### Outcome and Follow-Up

The outcome of interest was the diagnosis of incident seizure or epilepsy, defined by ICD-9-CM diagnostic codes 7803 or 345 in at least one inpatient claim or at least two emergency room or outpatient claims. In Taiwan's NHIRD, the sensitivity and specificity were 83.91 and 99.83%, respectively, for the claims diagnosis of epilepsy (17). Patients were followed from the index date until the occurrence of the outcome, mortality, disenrollment from National Health Insurance, or the end of the database (December 31, 2015), whichever came first.

### Statistical Analysis

Descriptive statistics were used to summarize the baseline characteristics in the study population. The continuous variables were described as means with SDs, and the categorical variables were described by number and proportions. The differences in the baseline characteristics between MCA and non-MCA groups were tested using a *t*-test for the continuous variables and the chi-square for the categorical variables. Next, the distribution of time to seizure or epilepsy was estimated using the Kaplan–Meier method. Then, the multivariable Cox proportional hazard model was used to identify the risk factors for developing seizures or epilepsy after an ischemic stroke, and the relative hazard in the MCA group has been estimated further and was compared with that of the non-MCA group by stratifying the risk factors. All significance levels were two-sided: *P* < 0.05. Statistical analyses have been performed using SAS software (version 9.4 for Windows; SAS Institute Inc., Cary, NC, USA).

We adopted the external adjustment method which was developed by Schneeweiss (18) to estimate the effects of relative risk adjusting unmeasured confounding factors. The limitations of this method were: (1) exploring only one external variable; (2) the external variables have to be a binary variable. Therefore, we grouped the NIHSS variable (NIHSS score: ≥16 vs. NIHSS score: ≤15) and Glasgow coma scale (GCS score: 3–13 vs. GCS score: 14–15) in two groups. We then performed two analyses: the adjusted relative risk of Glasgow coma scale score after adjusting NIHSS and the adjusted relative risk of urinary tract infection after adjusting NIHSS. The estimated relative risk for PSE in patients with NIHSS score ≥16 compared to NIHSS score ≤15 was 2.23 and we used the estimate as to the parameter for external adjustment.

## Results

### Characteristics of All Patients With Ischemic Stroke (MCA Infarct vs. Non-MCA Infarct)

In total, 2,769 patients with ischemic stroke were identified from the Hospital Stroke Registry and were successfully linked to the NHIRD. About 670 patients were excluded due to presenting with a prior stroke of any type within the previous 3 years, 61 patients were excluded due to prior seizures or epilepsy, and 200 patients were excluded due to prescriptions for antiepileptic drugs within the previous year. Of the remaining 1,838 patients, 774 and 1,064 were MCA infarct and non-MCA infarct, respectively (Figure 1).

The baseline characteristics of the patients are demonstrated in Table 1. Compared to patients with non-MCA infarct, those with MCA infarct were older (71.8 ± 12.2 vs. 69.8 ± 12.0, *P* < 0.001), presented with more atrial fibrillation (25.2 vs. 12.7%, *P* < 0.001) and prior warfarin use (8.5 vs. 4.8%, *P* < 0.001), less hyperlipidemia (43.9 vs. 51.2%, *P* = 0.002), and prior statin use (32.9 vs. 40.9%, *P* < 0.001). They exhibited higher NIHSS and mRS scores (both *P* < 0.001), were less likely to be alert on arrival (74.9 vs. 91.9%, *P* < 0.001), received more neurosurgeries (1.8 vs. 0.3%, *P* < 0.001), and exhibited more inhospital complications, namely, pneumonia (10.9 vs. 5.3%, *P* < 0.001) and hemorrhagic infarcts (4.7 vs. 1/0%, *P* < 0.001).

**Table 1:** Characteristics of all patients experiencing an ischemic stroke.

**Variable**	**MCAI**		**Non-MCAI**		***P* value**
	**n**	**%**	**n**	**%**	
	774	100	1,064	100	
*Demographics*					
Female	350	45.2	464	43.6	0.493
Age, years, mean (SD)	71.8	12.2	69.8	12.0	<0.001
Age group, years					<0.001
<60	123	15.9	200	18.8	
60–79	440	56.8	656	61.7	
≥80	211	27.3	208	19.5	
Length of follow-up (year), mean (SD)	5.2	2.6	5.3	2.5	0.298
Smoking	275	35.5	381	35.8	0.892
Education years	5.9	5.0	5.8	4.7	0.585
*Comorbidity*					
Hypertension	648	83.7	893	83.9	0.905
Diabetes mellitus	311	40.2	460	43.2	0.191
Hyperlipidemia	340	43.9	545	51.2	0.002
Atrial fibrillation	195	25.2	135	12.7	<0.001
Coronary artery disease	175	22.6	208	19.5	0.111
Dementia	70	9.0	76	7.1	0.137
Depression	43	5.6	48	4.5	0.137
*Medication use*					
Antiplatelet agents	638	82.4	906	85.2	0.116
Antidiabetic agents	291	37.6	412	38.7	0.624
Warfarin	66	8.5	51	4.8	0.001
Statin	255	32.9	435	40.9	<0.001
*Disease severity*					
NIHSS					<0.001
<8	422	54.5	861	80.9	
8–15	172	22.2	132	12.4	
≥16	180	23.3	71	6.7	
mRS					<0.001
1–2	293	37.9	649	61.0	
3–4	334	43.2	353	33.2	
5–6	147	19.0	62	5.8	
HbA1c, mg/dl					0.053
<7	314	40.6	473	44.5	
7–9	403	52.1	495	46.5	
≥10	57	7.4	96	9.0	
Systolic blood pressure, mmHg					0.346
<140	182	23.5	257	24.2	
140–180	379	49.0	546	51.3	
>180	213	27.5	261	24.5	
*Alert on arrival*	580	74.9	978	91.9	<0.001
*Neurosurgery*	14	1.8	3	0.3	<0.001
*Complication*					
Pneumonia	84	10.9	56	5.3	<0.001
Urinary tract infection	64	8.3	70	6.6	0.169
Hemorrhagic infarct	36	4.7	11	1.0	<0.001

*MCAI, middle cerebral artery infarct; NIHSS, National Institutes of Health Stroke Scale; mRS, Modified Rankin Scale; SD, Standard Deviation*.

### The Risk Factors for PSE in Patients With Ischemic Stroke (MCA Infarct and Other Risk Factors for PSE)

The risk factors for PSE in patients who experienced an ischemic stroke were investigated further using the multivariable Cox proportional hazard model. The unadjusted and adjusted hazard ratios (HRs) (95% CI) of MCA infarct for PSE were 2.48 (1.64–3.76) and 2.06 (1.33–3.19), respectively. Other factors significantly associated with PSE in the multivariable analysis [unadjusted HR (95% CI) and adjusted HR (95% CI)] included atrial fibrillation [2.65 (1.75–4.01) and 1.76 (1.06–2.91)], depression [2.24 (1.17–4.31) and 2.00 (1.02–3.91)], NIHSS scores of ≥ 16 [3.17 (1.99–5.05) and 3.21 (1.47–7.00)], and alert on arrival [1.6 (0.99–2.6) and 0.43 (0.21–0.88)] (Table 2).

**Table 2:** The risk factors for poststroke epilepsy in patients with ischemic stroke using the multivariable Cox proportional hazard model.

**Variable**	**Unadjusted HR**	**Adjusted HR**
	**(95% CI)**	**(95% CI)**
*Demographics*		
MCAI vs. non-MCAI	2.48 (1.64–3.76)	2.06 (1.33–3.19)
Male vs. female	0.69 (0.46–1.03)	0.99 (0.55–1.78)
Age, years	1.04 (1.02–1.06)	1.01 (0.99–1.04)
Length of ICU stay, days	1.05 (1.02–1.08)	1.04 (0.99–1.09)
Smoker vs. nonsmoker	0.88 (0.58–1.35)	1.34 (0.77–2.33)
Education years	0.92 (0.87–0.96)	0.94 (0.89–0.99)
*Comorbidity*		
Hypertension	1.83 (0.92–3.64)	1.67 (0.81–3.42)
Diabetes mellitus	0.75 (0.5–1.14)	0.67 (0.33–1.36)
Hyperlipidemia	0.82 (0.55–1.23)	1.24 (0.77–1.99)
Atrial fibrillation	2.65 (1.75–4.01)	1.76 (1.06–2.91)
Coronary artery disease	1.68 (1.09–2.6)	1.25 (0.78–2.01)
Dementia	0.89 (0.41–1.92)	0.74 (0.33–1.66)
Depression	2.24 (1.17–4.31)	2.00 (1.02–3.91)
*Medication use*		
Antiplatelet agents	1.26 (0.7–2.25)	1.00 (0.54–1.87)
Antidiabetic agents	0.94 (0.62–1.42)	1.33 (0.65–2.7)
Warfarin	2.15 (1.17–3.93)	1.30 (0.66–2.59)
Statin	0.61 (0.39–0.96)	0.56 (0.33–0.96)
*Disease severity*		
NIHSS		
<8	1.00	1.00
8–15	1.92 (1.15–3.2)	1.57 (0.88–2.8)
≥16	3.17 (1.99–5.05)	3.21 (1.47–7.00)
mRS		
1–2	1.00	1.00
3–4	1.78 (1.13–2.79)	1.08 (0.64–1.82)
5–6	2.84 (1.64–4.94)	0.68 (0.30–1.58)
HbA1C, mg/dl	0.99 (0.9–1.09)	1.00 (0.98–1.02)
Systolic blood pressure, mmHg	1.00 (0.99–1.01)	1.00 (0.99–1.01)
*Alert on arrival*	1.6 (0.99–2.6)	0.43 (0.21–0.88)
*Neurosurgery*	—^†^	—^†^
*Complication*		
Pneumonia	1.83 (1.00–3.35)	0.91 (0.44–1.86)
Urinary tract infection	2.67 (1.56–4.56)	1.55 (0.85–2.82)
Hemorrhagic infarct	2.38 (1.04–5.45)	0.87 (0.35–2.17)

† *The results are not shown owing to the small sample size*.

*MCAI, middle cerebral artery infarct; NIHSS, National Institutes of Health Stroke Scale; mRS, Modified Rankin Scale; HR, Hazard Ratio*.

### Stratified HRs for PSE (MCA Infarct vs. Non-MCA Infarct)

In the stratified analysis (Supplementary Figure 2), the trend of a positive association between MCA infarct and PSE was shown across subgroups of sex (male), age (60–79 years), hypertension, and use of antiplatelet agents. Similarly, disease severity (NIHSS, mRS, and nonalert on arrival) showed a positive trend between MCA infarct and PSE (*P* values for all interactions > 0.05).

### The Risk Factors for Developing PSE in Patients With MCA Infarct

The risk factors for developing PSE specifically in patients with MCA infarcts have been analyzed further. For patients with MCA infarcts, the disease severity [higher NIHSS (*P* = 0.002) and Glasgow coma scale scores (*P* = 0.006)], the presence of visual field defects (*P* = 0.034) and limb weakness (*P* = 0.005), urination control impairment (*P* = 0.019), and complications during hospitalization [urinary tract infection (*P* = 0.019), and hemorrhagic infarct (*P* = 0.05)] were associated with a higher risk for PSE development (Table 3).

**Table 3:** The risk factors for developing poststroke epilepsy in patients with MCA infarcts.

**Variable**	**Occurrence of poststroke epilepsy**				
	**Yes**		**No**		***P* value**
	***n***	**%**	***n***	**%**	
Total	62	100	712	100	
*Disease severity*					
NIHSS					0.002
<8	22	35.5	400	56.2	
8–15	15	24.2	157	22.1	
≥16	25	40.3	155	21.8	
mRS					
1–2					0.070
3–4	16	25.8	277	38.9	
5–6	29	46.8	305	42.8	
Glasgow coma scale					0.006
3–8	5	8.1	46	6.5	
9–13	25	40.3	163	22.9	
14–15	32	51.6	503	70.6	
Hemineglect	11	17.7	95	13.3	0.334
Visual field defect	21	33.9	157	22.1	0.034
Limb weakness, mean, SD^†^	5.6	3.8	4.1	5.0	0.005
Length of ICU stay, mean, SD (days)	3.2	7.0	1.4	4.1	0.055
Systolic blood pressure, mean, SD (mmHg)	162.4	32.3	162.9	31.9	0.910
HbA1C, mean, SD (mg/dl)	7.1	1.2	7.2	1.6	0.743
Urination control, mean, SD	6.5	4.3	7.7	3.9	0.019
*Alert on arrival*	44	71.0	536	75.3	0.452
*Neurosurgery*	0	0.0	14	2.0	0.308
*Complication*					
Pneumonia	9	14.5	75	10.5	0.337
Urinary tract infection	10	16.1	54	7.6	0.019
Hemorrhagic infarct	6	9.7	30	4.2	0.050

† *Presented as the sum of items 5a, 5b, 6a, and 6b in the National Institutes of Health Stroke Scale score (range 0–16)*.

*NIHSS, National Institutes of Health Stroke Scale; mRS, Modified Rankin Scale; SD, Standard Deviation*.

### The Incidence Curves for Developing PSE in Patients With MCA Infarcts vs. Non-MCA Infarcts

**Figure 2:**
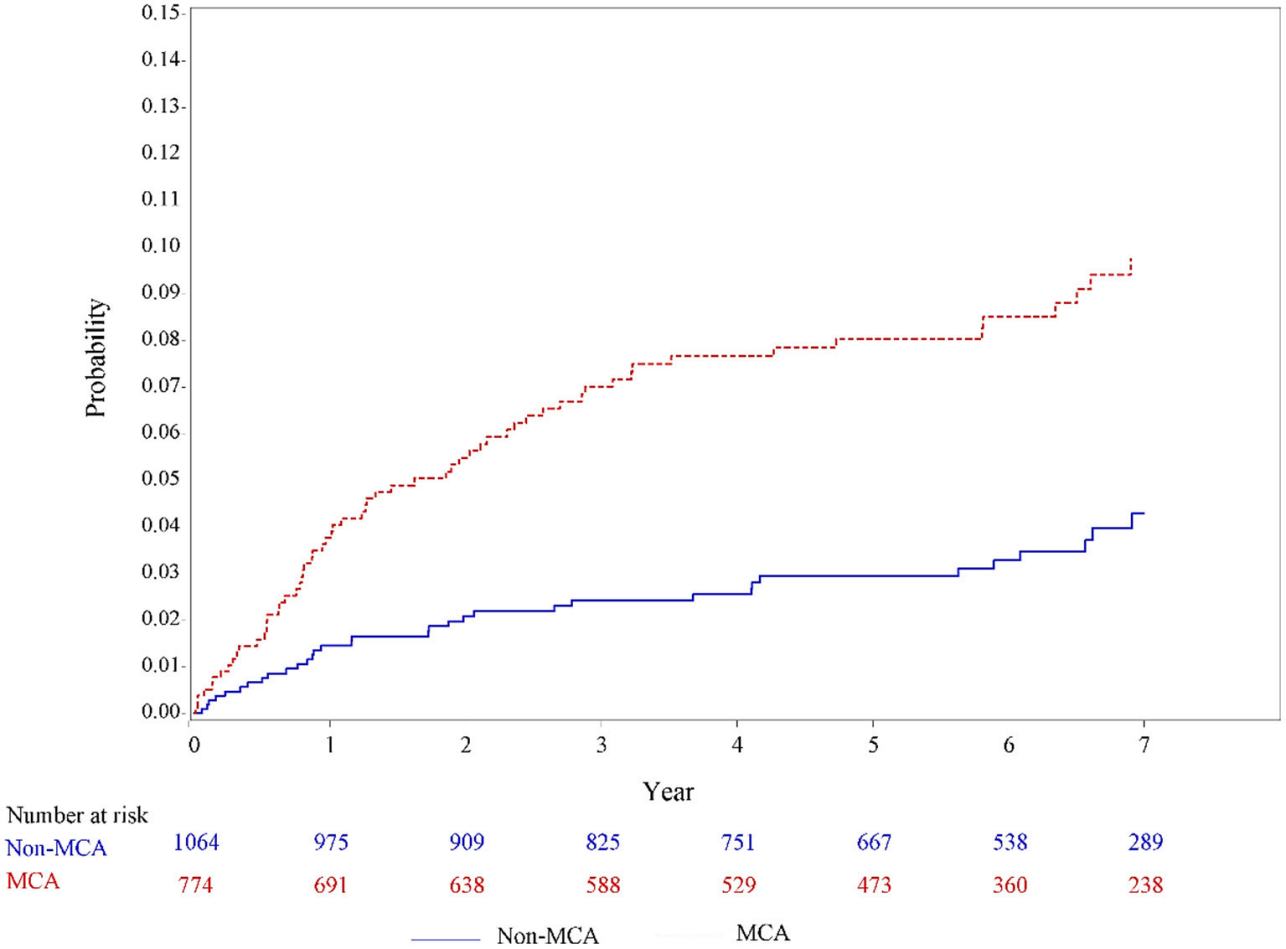
Unadjusted incidence curves for developing poststroke epilepsy in patients with MCA infarcts and non-MCA infarcts based on the Hospital Stroke Registry. MCA, middle cerebral artery.

In the univariable analysis using the Kaplan–Meier method (Figure 2), a significantly higher incidence of PSE was observed among patients with MCA infarcts than those patients with non-MCA infarcts (incidence rate, 15.5 vs. 6.2 per 1,000 person-year; *P* = 0.0012).

## Discussion

In this study, all patients with ischemic stroke (MCA infarct vs. non-MCA infarct) were first characterized, and the risk factors for PSE in all patients were investigated; then, HRs for PSE were stratified (MCA infarct vs. non-MCA infarct); finally, the risk factors for developing PSE in patients with MCA infarcts have been specifically evaluated. Furthermore, in our survival analysis, the incidence curves for the development of PSE in patients with MCA infarcts vs. non-MCA infarcts have been depicted. In addition to establishing the higher risk of PSE following an MCA infarct as compared to a non-MCA infarct, the important clinical risk factors associated with PSE in patients with MCA infarcts were identified: the disease severity [higher NIHSS and Glasgow coma scale scores, the presence of visual field defects and limb weakness, urination control impairment, and complications during hospitalization (urinary tract infection and hemorrhagic infarcts)].

The MCA is the most common artery involved in an acute stroke (9). It branches directly from the internal carotid artery and consists of four main branches: M1, M2, M3, and M4. They provide important blood supply to parts of the frontal, temporal, and parietal lobes of the brain, and deeper structures, namely, the caudate, internal capsule, and thalamus (19). Studies reported that large events involving the middle and superior temporal gyri are particularly associated with the development of poststroke late seizures (20), supplied by the MCA (21). The lateral and inferior frontal gyri and anterior lateral part of the parietal lobe, supplied by the MCA, are also associated with a high risk of seizures (6, 22, 23). It is thus conceivable that due to the involvement of the temporal and frontal lobes, this area may be more susceptible to epileptogenesis after stroke than other brain areas.

In this study, patients with MCA infarcts exhibited more atrial fibrillation as compared to those with non-MCA infarcts, which was compatible with the findings of earlier studies showing atrial fibrillation to be associated with MCA infarcts (24), suggesting that statins may be beneficial in atrial fibrillation-related strokes (25). Moreover, less hyperlipidemia and prior statin use were found in patients with MCA infarcts. The findings of another study also suggested that statin use before the onset of nonlacunar MCA infarction is associated with a smaller infarct volume (26). Furthermore, the overall MCA infarcts were associated with higher NIHSS and mRS scores, consistent with the initial presentation, where these patients were less likely to be alert on arrival. Meanwhile, to infer that patients with MCA infarcts received more neurosurgeries and exhibited more in-hospital complications, namely, pneumonia, urinary tract infections, and hemorrhagic infarcts, would be reasonable.

Regarding the overall risk factors for PSE in patients experiencing an ischemic stroke, MCA infarcts were identified to exhibit the highest adjusted HR among all the variables. Atrial fibrillation, NIHSS scores of ≥16, alert on arrival, and depression were the other risk factors associated with PSE. The stratified specific comparison of the MCA infarct vs. non-MCA infarct groups on HR showed, in addition to an overall higher risk of PSE for the MCA infarct groups, a positive trend of an association was found between MCA infarct and PSE in some subgroups, namely, sex (male), age (60–79 years), hypertension, and using of antiplatelet agents, disease severity (NIHSS, mRS, and nonalert on arrival). The reason that the other risk factors did not show a strong positive trend is probably due to the strong HR of MCA infarct itself in this stratified analysis. The above findings suggested that, compared to non-MCA infarcts, MCA infarcts exhibited a higher HR for PSE.

In patients with MCA infarcts, specific risk factors were identified. Disease severity, as expected, could represent brain damage and important risk factors that could predict PSE. Similarly, the presence of visual field defects, limb weakness, and urination control impairment all represented significant compromises in and neuronal damage to MCA vascular territory, namely, the temporal, parietal, and frontal territories. In another study specifically focusing on collateral flow in acute MCA infarcts (27), visual field defects were independently associated with poor collateral status in their multiple logistic regression. Similarly, visual field defects were associated with poor collateral status in another study (28). Poor collateral recruitment in MCA blood flow velocity may be related to seizures (29). In addition, visual field defects may represent a marker of stroke cortical localization, which has extensively been associated with poststroke epilepsy in previous studies (5).

Depression has been regarded as the peri-injury exposome and risk of epileptogenesis after cerebral stroke (6), which is consistent with the findings in our study in predicting overall PSE, but not specifically in MCA infarcts. In addition, urinary incontinence has been identified as a predictor of death and severe disability in stroke (30), and incontinence at stroke presentation is significantly associated with PSE (5). Urinary tract infections during hospitalization should be noted as an important risk factor for PSE. Although urinary tract infections have not been associated with a worsened outcome (31), the risk of PSE could significantly enhance the care burden. To our knowledge, this is the first study indicating that urination control and urinary tract infections are the main risk factors for developing PSE in patients with MCA infarcts. Whether urinary tract infection in patients with MCA infarcts would lead to PSE is worth further investigation.

Interestingly, one study proposing another scoring system for PSE prediction did not include the stroke location as an important variable (32). Nevertheless, one study proposed a prognostic model for predicting poststroke late seizures, in which the parameters were stroke severity, large-artery atherosclerotic etiology, early seizures, cortical involvement, and MCA territory involvement (12). In this study, MCA involvement led to a high HR in the univariable analysis, but not in the final multivariable Cox proportional hazards model. As the multivariate model included the two independent variables “cortical involvement” and “early seizures,” the significance of MCA infarct was less clear (12) and it suggests “cortical involvement” plays a more important role than the MCA infarct variable itself in PSE. Of note, the reliability of this prognostic model needs further verification (33). In our study, as our study mainly focuses on patients with MCA infarct, which included all the territories, we did not specify the extent of cortical involvement. The significance of MCA-infarct on PSE was clearly established. Furthermore, as to the early seizures, in our current prospective database, the number of early seizures was too low, which was deemed not to be able to generate enough statistical power.

Although the observations were retrospective, this study was based on prospectively registered patients with stroke who were admitted to National Cheng Kung University Hospital and Ditmanson Medical Foundation Chia-Yi Christian Hospital. The data collected in this stroke registry were prospectively recorded. Our study presents several additional limitations. The HR of acute seizure complications during hospitalization was not assessed precisely due to the small sample size. Performing long-term EEG monitoring on every patient with stroke is not practically feasible. Another limitation was the fact that the HR of neurosurgical intervention in PSE could not be evaluated due to the small sample size. Although patients who require neurosurgical procedures during the acute stage of a stroke typically represent a higher degree of severity, neurosurgical interventions, including hemicraniectomies, still require randomized clinical trials to prove their effects on functional recovery, especially in patients over 60 years of age and those who are severely disabled (34, 35). Besides, we did not perform a multivariable regression model to assess the factors associated with PSE in the MCA infarct group because of the insufficient number of PSE cases among patients with MCA infarcts. Alternatively, we adopted the external adjustment method to estimate the effects of relative risk adjusting unmeasured confounding factors in Supplementary Table 1. The risk of PSE was increased in lower Glasgow coma scale score (3–13) as compared to higher Glasgow coma scale score (14–15), and in patients with urinary tract infection as compared to patients without urinary tract infection. Further studies using other databases with a large sample size are still needed to verify the findings.

Regarding the effects of acute treatments for stroke (thrombolysis and thrombectomy), due to the extremely small number of patients, we did not verify the effects of acute treatments on PSE. Furthermore, according to the current database, we could neither differentiate the data based on the tracts of MCA occlusion (M1-M4) nor analyze the tandem occlusion stroke (MCA + ICA) in the MCA cohort. In addition, according to the database, at the current stage, we could only compare MCA infarcts with non-MCA infarcts (total); we could not differentiate between the subgroups in patients with non-MCA (e.g., patients with vertebrobasilar infarct). Whether different territories of MCA would carry the different risks of PSE is worth further investigation. Lastly, whether the results can be generalized to other racial/ethnic groups is unclear given that stroke and its complications may not be totally similar among different ethnic groups (8, 36).

## Conclusions

This study on a large dataset linking stroke registry and an administrative claims database established the higher risk of PSE and identified the important distinct clinical risk factors associated with PSE in patients with MCA infarcts: disease severity [higher NIHSS and Glasgow coma scale scores, presence of visual field defects and limb weakness, urination control impairment, and complications during hospitalization (urinary tract infections and hemorrhagic infarcts)]. Increased awareness and early management of these risk factors in patients with MCA infarcts may be important in PSE prevention and thus should be given attention.

## Data Availability Statement

The data analyzed in this study was obtained from the National Health Insurance Research Database (NHIRD), the following licenses/restrictions apply: these datasets were provided by the Ministry of Health and Welfare (MOHW) and are maintained by the Health and Welfare Data Science Center (HWDC) in Taipei City, Taiwan. Requests to access these datasets should be directed to Chin-Wei Huang, huangcw@mail.ncku.edu.tw.

## Ethics Statement

The studies involving human participants were reviewed and approved by Institutional Review Board of National Cheng Kung University Hospital (IRB No. A-ER-108-563). Written informed consent for participation was not required for this study in accordance with the national legislation and the institutional requirements.

## Author Contributions

C-YH, C-CS, EL, Y-SC, T-HH, Y-HK, C-HC, S-FS, and C-WH: substantial contributions to the conception or design of the work or the acquisition, analysis or interpretation of data for the work, provide approval for publication of the content, and agree to be accountable for all aspects of the work in ensuring that questions related to the accuracy or integrity of any part of the work are appropriately investigated and resolved. C-YH and C-WH: drafting the work or revising it critically for important intellectual content. All authors contributed to the article and approved the submitted version.

## Funding

This research was supported in part by grants from the Ministry of Science and Technology, Taiwan (107-2314-B-006-018-, 107-2320-B-006-019-, 108-2320-B-006-023-, and 109-2314-B-006-034 -MY3 to C-WH). The funding bodies did not play any role in the design of the study and collection, analyses, and interpretation of data or the writing of the manuscript.

## Conflict of Interest

The authors declare that the research was conducted in the absence of any commercial or financial relationships that could be construed as a potential conflict of interest.

## Publisher's Note

All claims expressed in this article are solely those of the authors and do not necessarily represent those of their affiliated organizations, or those of the publisher, the editors and the reviewers. Any product that may be evaluated in this article, or claim that may be made by its manufacturer, is not guaranteed or endorsed by the publisher.

## Supplementary Material

Supplementary Figure 1 Study design. PSE, poststroke epilepsy; NHIRD, National Health Insurance Research Database.

Supplementary Figure 2 The stratified hazard ratios for poststroke epilepsy in patients with MCAI as compared to non-MCAI. — Owing to privacy protection, the variables were masked as the number of cells was fewer or the hazard limit cannot be estimated due to the small sample size. ^*^The incidence was defined as the number of cases divided by the number of stroke patients per 1,000 person-years. MCAI, middle cerebral artery infarct; NIHSS, NIH Stroke Scale; mRS, Modified Rankin Scale.

Supplementary Table 1 External adjustment for estimate the effects of relative risk adjusting unmeasured confounding factors. ARR, apparent exposure relative risk; P_C0_, prevalence of confounder in the unexposed; P_C1_, prevalence of confounder in the exposed; RR_CD_, association between confounder and diseases outcome; RR_adj_, adjusted exposure relative risk.

## References

[1] Tanaka T Ihara M. Post stroke epilepsy. Neurochem Int. (2017) 107:219–28. 10.1016/j.neuint.2017.02.002 28202284

[2] Shetty AK. Prospects of levetiracetam as a neuroprotective drug against status epilepticus, traumatic brain injury, and stroke. Front Neurol. (2013) 4:172. 10.3389/fneur.2013.00172 24204362 PMC3816384

[3] Huang CW Saposnik G Fang J Steven DA Burneo JG. Influence of seizures on stroke outcomes: a large multicenter study. Neurology. (2014) 82:768–76. 10.1212/WNL.0000000000000166 24489133

[4] Awada A Omojola MF Obeid T. Late epileptic seizures after cerebral infarction. Acta Neurol Scand. (1999) 99:265–8. 10.1111/j.1600-0404.1999.tb00674.x 10348154

[5] Graham NS Crichton S Koutroumanidis M Wolfe CD Rudd AG. Incidence and associations of poststroke epilepsy: the prospective South London Stroke Register. Stroke. (2013) 44:605–11. 10.1161/STROKEAHA.111.000220 23370202

[6] Pitkanen A Roivainen R Lukasiuk K. Development of epilepsy after ischaemic stroke. Lancet Neurol. (2016) 15:185–97. 10.1016/S1474-4422(15)00248-3 26597090

[7] So EL Annegers JF Hauser WA O'Brien PC Whisnant JP. Population-based study of seizure disorders after cerebral infarction. Neurology. (1996) 46:350–5. 10.1212/WNL.46.2.350 8614493

[8] Naylor J Churilov L Johnstone B Guo R Xiong Y Koome M. The association between atrial fibrillation and poststroke seizures is Influenced by ethnicity and environmental factors. J Stroke Cerebrovasc Dis. (2018) 27:2755–60. 10.1016/j.jstrokecerebrovasdis.2018.05.044 30037649

[9] Ng YS Stein J Ning M Black-Schaffer RM. Comparison of clinical characteristics and functional outcomes of ischemic stroke in different vascular territories. Stroke. (2007) 38:2309–14. 10.1161/STROKEAHA.106.475483 17615368

[10] Ferlazzo E Gasparini S Beghi E Sueri C Russo E Leo A. Epilepsy in cerebrovascular diseases: Review of experimental and clinical data with meta-analysis of risk factors. Epilepsia. (2016) 57:1205–14. 10.1111/epi.13448 27381481

[11] De Carolis P D'Alessandro R Ferrara R Andreoli A Sacquegna T Lugaresi E. Late seizures in patients with internal carotid and middle cerebral artery occlusive disease following ischaemic events. J Neurol Neurosurg Psychiatry. (1984) 47:1345–7. 10.1136/jnnp.47.12.1345 6439825 PMC1028146

[12] Galovic M Döhler N Erdélyi-Canavese B Felbecker A Siebel P Conrad J. Prediction of late seizures after ischaemic stroke with a novel prognostic model (the SeLECT score): a multivariable prediction model development and validation study. Lancet Neurol. (2018) 17:143–52. 10.1016/S1474-4422(17)30404-0 29413315

[13] Hsieh CY Su CC Shao SC Sung SF Lin SJ Kao Yang YH. Taiwan's National Health Insurance Research Database: past and future. Clin Epidemiol. (2019) 11:349–58. 10.2147/CLEP.S196293 31118821 PMC6509937

[14] Hsieh FI Lien LM Chen ST Bai CH Sun MC Tseng HP. Get with the guidelines–stroke performance indicators: surveillance of stroke care in the Taiwan Stroke Registry: get with the guidelines–stroke in Taiwan. Circulation. (2010) 122:1116–23. 10.1161/CIRCULATIONAHA.110.936526 20805428

[15] Hsieh CY Wu DP Sung SF. Registry-based stroke research in Taiwan: past and future. Epidemiol Health. (2018) 40:e2018004. 10.4178/epih.e2018004 29421864 PMC5847969

[16] Lai ECC Man KKC Chaiyakunapruk N Cheng CL Chien HC Chui CSL. Brief report: databases in the Asia-Pacific region: the potential for a distributed network approach. Epidemiology. (2015) 26:815–20. 10.1097/EDE.0000000000000325 26133022

[17] Chen CC Chen LS Yen MF Chen HH Liou HH. Geographic variation in the age- and gender-specific prevalence and incidence of epilepsy: analysis of Taiwanese National Health Insurance-based data. Epilepsia. (2012) 53:283–90. 10.1111/j.1528-1167.2011.03332.x 22126307

[18] Schneeweiss S. Sensitivity analysis and external adjustment for unmeasured confounders in epidemiologic database studies of therapeutics. Pharmacoepidemiol Drug Saf. (2006) 15:291–303. 10.1002/pds.1200 16447304

[19] Shapiro M Raz E Nossek E Chancellor B Ishida K Nelson PK. Neuroanatomy of the middle cerebral artery: implications for thrombectomy. J Neurointerv Surg. (2020) 12:768–73. 10.1136/neurintsurg-2019-015782 32107286

[20] Heuts-van Raak L Lodder J Kessels F. Late seizures following a first symptomatic brain infarct are related to large infarcts involving the posterior area around the lateral sulcus. Seizure. (1996) 5:185–94. 10.1016/S1059-1311(96)80034-3 8902919

[21] De Long WB. Anatomy of the middle cerebral artery: the temporal branches. Stroke. (1973) 4:412–8. 10.1161/01.STR.4.3.412 4713030

[22] Kellinghaus C Luders HO. Frontal lobe epilepsy. Epileptic Disord. (2004) 6:223–39. 15634619

[23] Salanova V. Parietal lobe epilepsy. Handb Clin Neurol. (2018) 151:413–25. 10.1016/B978-0-444-63622-5.00021-8 29519472

[24] Assarzadegan F Tabesh H Shoghli A Ghafoori Yazdi M Tabesh H Daneshpajooh P. Relation of stroke risk factors with specific stroke subtypes and territories. Iran J Public Health. (2015)44:1387–94. 26576352 PMC4644584

[25] Lee MJ Bang OY Kim SJ Kim GM Chung CS Lee KH. Role of statin in atrial fibrillation-related stroke: an angiographic study for collateral flow. Cerebrovasc Dis. (2014) 37:77–84. 10.1159/000356114 24457535 PMC4157914

[26] Shook SJ Gupta R Vora NA Tievsky AL Katzan I Krieger DW. Statin use is independently associated with smaller infarct volume in nonlacunar MCA territory stroke. J Neuroimaging. (2006) 16:341–6. 10.1111/j.1552-6569.2006.00061.x 17032384

[27] Hwang J Lee MJ Chung JW Bang OY Kim GM Chung CS. NIHSS sub-item scores predict collateral flow in acute middle cerebral artery infarction. Interv Neuroradiol. (2018) 24:678–83. 10.1177/1591019918788056 29991309 PMC6259331

[28] Nannoni S Sirimarco G Cereda CW Lambrou D Strambo D Eskandari A. Determining factors of better leptomeningeal collaterals: a study of 857 consecutive acute ischemic stroke patients. J Neurol. (2019) 266:582–8. 10.1007/s00415-018-09170-3 30610425

[29] Naylor AR Evans J Thompson MM London NJ Abbott RJ Cherryman G. Seizures after carotid endarterectomy: hyperperfusion, dysautoregulation or hypertensive encephalopathy. Eur J Vasc Endovasc Surg. (2003) 26:39–44. 10.1053/ejvs.2002.1925 12819646

[30] Brittain KR Peet SM Castleden CM. Stroke and incontinence. Stroke. (1998) 29:524–8. 10.1161/01.STR.29.2.524 9472900

[31] Rohweder G Ellekjær H Salvesen Ø Naalsund E Indredavik B. Functional outcome after common poststroke complications occurring in the first 90 days. Stroke. (2015) 46:65–70. 10.1161/STROKEAHA.114.006667 25395415

[32] Chi NF Kuan YC Huang YH. Development and validation of risk score to estimate 1-year late poststroke epilepsy risk in ischemic stroke patients. Clin Epidemiol. (2018) 10:1001–11. 10.2147/CLEP.S168169 30174459 PMC6110266

[33] Finsterer J. The SeLECT score is inappropriate to predict post-stroke epilepsy. Lancet Neurol. (2018) 17:106–7. 10.1016/S1474-4422(17)30460-X 29413306

[34] Alexander P Heels-Ansdell D Siemieniuk R Bhatnagar N Chang Y Fei Y. Hemicraniectomy vs. medical treatment with large MCA infarct: a review and meta-analysis. BMJ Open. (2016) 6:e014390. 10.1136/bmjopen-2016-014390 27884858 PMC5168488

[35] Wei H Jia FM Yin HX Guo ZL. Decompressive hemicraniectomy vs. medical treatment of malignant middle cerebral artery infarction: a systematic review and meta-analysis. Biosci Rep. (2020) 40:BSR20191448. 10.1042/BSR20191448 31854446 PMC6944664

[36] Boehme AK Esenwa C Elkind MS. Stroke risk factors, genetics, and prevention. Circ Res. (2017) 120:472–95. 10.1161/CIRCRESAHA.116.308398 28154098 PMC5321635

