# Supplementary figures and images for: Risk of Incident Epilepsy After a Middle Cerebral Artery Territory Infarction
Source: Front Neurol. 2022 Mar 3;13:765969. doi: 10.3389/fneur.2022.765969 (PMC8930196; doi:10.3389/fneur.2022.765969)

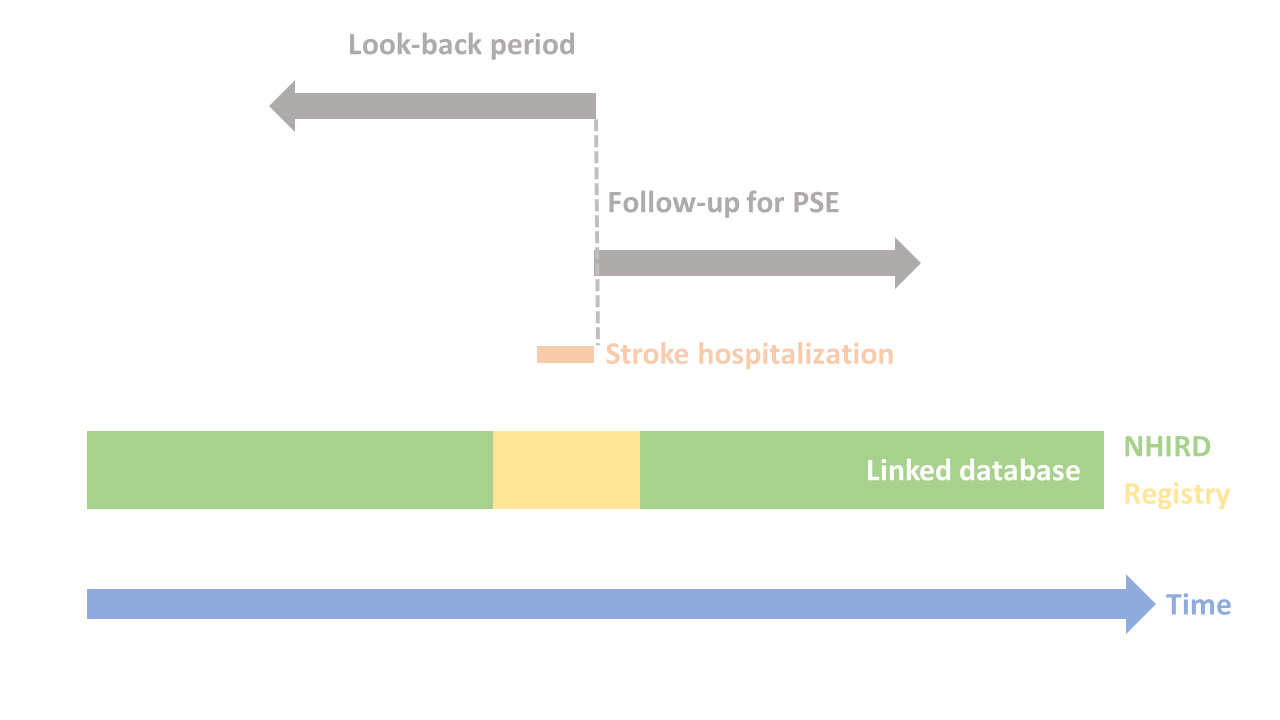

Supplement: Supplementary Figure 1 — Study design. PSE, poststroke epilepsy; NHIRD, National Health Insurance Research Database. [file Image_1.TIF]

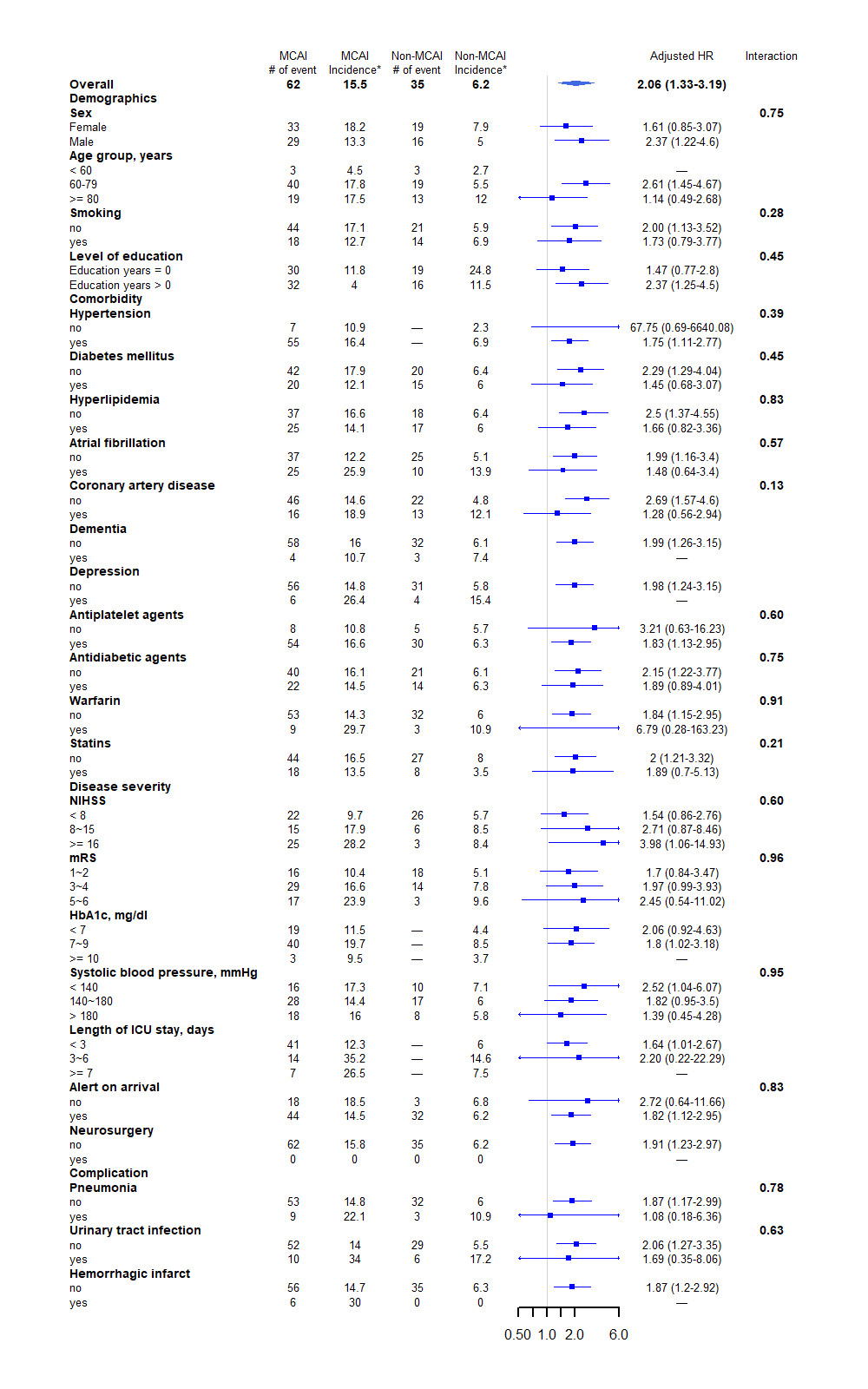

Supplement: Supplementary Figure 2 — The stratified hazard ratios for poststroke epilepsy in patients with MCAI as compared to non-MCAI. — Owing to privacy protection, the variables were masked as the number of cells was fewer or the hazard limit cannot be estimated due to the small sample size. *The incidence was defined as the number of cases divided by the number of stroke patients per 1,000 person-years. MCAI, middle cerebral artery infarct; NIHSS, NIH Stroke Scale; mRS, Modified Rankin Scale. [file Image_2.TIFF]
