# Supplementary material for: Risk of Incident Epilepsy After a Middle Cerebral Artery Territory Infarction
Source: Front Neurol. 2022 Mar 3;13:765969. doi: 10.3389/fneur.2022.765969 (PMC8930196; doi:10.3389/fneur.2022.765969)
Supplement: Supplementary Table 1 — External adjustment for estimate the effects of relative risk adjusting unmeasured confounding factors. ARR, apparent exposure relative risk; PC0, prevalence of confounder in the unexposed; PC1, prevalence of confounder in the exposed; RRCD, association between confounder and diseases outcome; RRadj, adjusted exposure relative risk. [file Table_1.DOCX]

**Supplementary Table 1.** External adjustment for estimate of the effects of relative risk adjusting unmeasured confounding factors.

| Variable | Adjusted  variable | **ARR** | P_C0_,  (NIHSS:≦15) | P_C1_  (NIHSS:≧16) | RR_CD_  (NIHSS:≧16  vs  NIHSS:≦15) | **RR_adj_** |
| --- | --- | --- | --- | --- | --- | --- |
| Glasgow coma scale  (3-13 vs. 14-15) | NIHSS | 1.1 | 0.77 | 0.23 | 2.23 | 1.66 |
| Urinary tract infection  (Yes vs. No) | NIHSS | 1.9 | 0.77 | 0.23 | 2.23 | 2.87 |

ARR: apparent exposure relative risk in patients with Glasgow coma scale score in range of 3-13 compared to range of 14-15 or in patients with urinary tract infection compared to without urinary tract infection.

P_C0_: prevalence of confounder in the unexposed. The confounder in this situation was stroke severity (NIHSS) and the unexposed was NIHSS score≦15.

P_C1_: prevalence of confounder in the exposed. The confounder in this situation was stroke severity (NIHSS) and the exposed was NIHSS score≧16.

RR_CD_: association between confounder and diseases outcome. In this situation, RR_CD_ was relative risk for PSE in patients with NIHSS score≧16 compared to NIHSS score≦15.

RR_adj_: adjusted exposure relative risk in patients with Glasgow coma scale in range of 3-13 compared to range of 14-15 or in patients with urinary tract infection compared to without urinary tract infection.
